# Mapping Assessment Approaches to Midwife-Led Perinatal Nutrition Counselling: A Scoping Review of Existing Instruments, Competency Frameworks, and Measurement Gaps

**DOI:** 10.3390/nursrep16090327

**Published:** 2026-09-07

**Authors:** Artemisia Kokkinari, Evangelia Antoniou, Dimitrios Iatrakis, Anastasia Bothou, Athina Diamanti, Evangelia Leontitsi, Maria Dagla

**Affiliations:** 1 Department of Midwifery, School of Health & Care Sciences, University of West Attica, 12243 Aigaleo, Greece; 2 Laboratory of Midwifery Care During Antenatal and Post Natal Period—Breastfeeding—L.M.C.A.P.-BF., University of West Attica, 12243 Aigaleo, Greece; 3 Department of Electrical and Computer Engineering, National Technical University of Athens, Zografou Campus, 9 Iroon Polytechniou Street, 15780 Zografou, Greece

**Keywords:** nutrition counselling, midwifery, perinatal care, assessment tools, patient-reported experience measures, scoping review

## Abstract

**Background:** Nutrition counselling during the perinatal period is an important component of midwifery care and health promotion. Existing competency frameworks recognize nutrition-related education and counselling as part of midwifery practice, while several studies have assessed midwives’ nutrition knowledge, attitudes, confidence, and self-reported practices. However, it remains unclear to what extent existing assessment approaches capture the quality of nutrition counselling as experienced by women receiving midwife-led care. **Objective:** This scoping review aimed to map existing tools, questionnaires, competency frameworks, checklists, and other assessment approaches related to nutrition counselling, nutrition education, and nutrition-related competencies in midwifery and perinatal care; to compare the measurement domains represented across these approaches; and to identify remaining measurement gaps. **Methods:** The review was conducted according to established scoping review methodology and reported in accordance with PRISMA-ScR. PubMed/MEDLINE, Scopus, Web of Science Core Collection, CINAHL, and Embase were searched from database inception to 1 July 2026 using two complementary search strategies targeting both nutrition-related assessment approaches and patient-experience/counselling-quality concepts. Eligible sources included studies or documents describing instruments, competency frameworks, questionnaires, screening tools, checklists, or other assessment approaches related to nutrition counselling within midwifery or perinatal care. Data were charted according to assessment type, target population, intended purpose, domains assessed, development methodology, validation evidence, and relevance to midwife-led nutrition counselling, and were synthesized descriptively and conceptually across assessment domains. **Results:** The database searches identified 186 records, of which 42 duplicates were removed, leaving 144 records for title and abstract screening. Thirty-four reports underwent full-text assessment, of which 25 were excluded, resulting in nine included reports. These reports represented a smaller number of distinct assessment tools, frameworks, and approaches because three reports evaluated or applied the same underlying instrument, the FIGO Nutrition Checklist. The included evidence comprised nutritional-risk screening, provider-focused knowledge-, attitude-, and practice-based assessment, observational approaches to counselling delivery, and a professional competency framework. Cross-cutting synthesis identified four conceptually distinct measurement levels: nutritional risk and dietary status, professional knowledge and competence, counselling delivery and implementation, and women’s experience of nutrition counselling. No eligible nutrition-specific patient-reported experience measure was identified, leaving the intersection between nutrition-specific content and woman-reported counselling experience insufficiently represented. **Conclusions:** Existing approaches provide fragmented assessment of nutrition-related care and do not comprehensively capture women’s experiences of midwife-led nutrition counselling. Further research should determine whether existing measures can be adapted or combined or whether a new patient-reported experience measure (PREM) is warranted.

## 1. Introduction

Nutrition counselling has become an integral component of high-quality perinatal care, reflecting the growing recognition that maternal nutrition influences both immediate pregnancy outcomes and the long-term health of mothers and their offspring [1]. Beyond the prevention of nutritional deficiencies, effective nutrition counselling plays a fundamental role in supporting healthy dietary behaviours, optimizing gestational weight gain, improving maternal metabolic health, and promoting favourable maternal and neonatal outcomes [2]. Accordingly, international organizations increasingly recommend that evidence-based nutrition counselling should be routinely incorporated into antenatal care rather than being reserved only for women identified as nutritionally at risk [3]. However, translating nutritional recommendations into routine clinical practice requires more than the availability of evidence-based guidelines. Effective nutrition counselling should be individualized, evidence-informed, and responsive to women’s clinical, cultural, and socioeconomic circumstances to facilitate meaningful behavioural change rather than simply transferring nutritional information [4].

Midwives are uniquely positioned to deliver nutrition counselling throughout the perinatal period because they provide continuous, woman-centred care across pregnancy, birth, and the postpartum period. Through repeated contacts and the development of trusting relationships, midwives have the opportunity to assess women’s nutritional needs, provide individualized dietary advice, reinforce healthy behaviours, and support informed decision-making as pregnancy progresses [5]. Effective midwife-led nutrition counselling involves not only the provision of accurate information but also its appropriate communication and individualization to support sustainable behavioural change [4,6]. Reflecting this broader role, international competency frameworks recognize nutrition counselling, health promotion, and preventive care as core components of midwifery practice and emphasize their integration into routine maternity care [6,7,8].

As recognition of the importance of nutrition within maternity care has increased, a variety of frameworks, questionnaires, screening tools, and assessment approaches have been introduced to evaluate different aspects of nutrition-related care and professional practice. Existing approaches can be broadly categorized into four groups. First, international competency frameworks define the knowledge, skills, and professional behaviours expected of midwives in delivering nutrition-related care and health promotion [5,6,7]. Second, knowledge-, attitude-, and practice-based (KAP) questionnaires primarily assess midwives’ nutrition knowledge, confidence, self-reported practices, and perceived barriers to providing nutrition counselling [9,10]. Third, nutritional screening tools, such as the FIGO Nutrition Checklist, are designed to identify women’s dietary risks and facilitate nutrition-related discussions during routine antenatal care rather than evaluate the counselling process itself [4]. Finally, structured observational approaches have been used to document whether and how predefined nutrition-counselling activities were delivered during clinical encounters, thereby providing information on counselling delivery and implementation from an observational perspective [11]. The existing approaches identified in the literature differ considerably with respect to their objectives, target populations, measurement domains, and intended applications. Their principal characteristics, intended purposes, and key limitations are summarized in Table 1.

The available assessment landscape encompasses distinct constructs and measurement purposes, including nutritional risk, professional competence and knowledge, and counselling delivery or implementation [4,5,6,7,8,9,10,11]. Within the nutrition-specific literature, however, patient-reported assessment of women’s experience of the counselling process appears to represent a separate and comparatively underdeveloped measurement area. For the purposes of the present review, the quality of midwife-led perinatal nutrition counselling from the woman’s perspective was conceptualized as an evidence-informed and woman-centred counselling process characterized by clear communication, individualized and practically applicable guidance, responsiveness to women’s clinical and personal circumstances, involvement in decision-making, continuity of support, and a supportive and non-judgmental therapeutic relationship [4,5,6,7,8]. This working definition was used to guide the interpretation of the measurement landscape rather than as a formally validated definition or predetermined measurement model. Accordingly, the patient-reported construct of interest in this review was counselling experience rather than counselling outcomes: it concerned how women experienced the communication, individualization, responsiveness, involvement in decision-making, continuity, and interpersonal support provided during nutrition counselling, rather than satisfaction alone, nutrition knowledge acquired, self-efficacy, subsequent dietary behaviour, or maternal and neonatal clinical outcomes. This gap is distinct from the broader literature on maternity care quality and patient-reported experience, because the present review focuses specifically on how nutrition counselling is operationalized and measured within midwifery and perinatal care. Rather than asking only whether a patient-reported measure exists, the review examines how the current measurement landscape is distributed across nutritional risk, professional competence, counselling delivery, and women’s experience, and which dimensions of woman-centred nutrition counselling remain insufficiently represented.

Against this background, the present scoping review aimed to systematically identify and map the existing instruments, competency frameworks, questionnaires, screening tools, checklists, and other assessment approaches related to nutrition counselling in midwifery and perinatal care. Specifically, the review sought to examine which dimensions of nutrition-related care are currently evaluated, how these constructs are operationalized, and whether existing approaches adequately capture women’s experiences of the quality of midwife-led perinatal nutrition counselling [4,5,6,7,8,9,10,11]. Beyond cataloguing existing approaches, the review sought to identify areas of conceptual convergence and divergence across assessment purposes and measurement domains and to determine which dimensions of woman-centred nutrition counselling remain insufficiently operationalized. This synthesis is intended to provide a conceptual basis for subsequent research examining whether existing measures can be adapted or combined, or whether development of a new patient-reported instrument is warranted, following internationally accepted methodological standards for instrument development and content validation [12,13].

## 2. Materials and Methods

### 2.1. Study Design

The present study was conducted as a scoping review to systematically identify and map the available evidence on instruments, competency frameworks, questionnaires, screening tools, checklists, and other assessment approaches related to nutrition counselling in midwifery and perinatal care. A scoping review methodology was considered the most appropriate approach because the objective of this study was to comprehensively examine the extent, characteristics, and nature of the available evidence, identify how nutrition counselling has been assessed across different settings, and explore existing measurement gaps rather than evaluate intervention effectiveness or estimate pooled effects.

The review methodology followed the Joanna Briggs Institute (JBI) methodology for scoping reviews, which builds upon the original methodological framework proposed by Arksey and O’Malley [14] and subsequent methodological refinements by Levac et al. [15]. The review was reported in accordance with the Preferred Reporting Items for Systematic Reviews and Meta-Analyses extension for Scoping Reviews (PRISMA-ScR) to ensure methodological rigour, transparency, reproducibility, and completeness of reporting [14,15,16,17].

A review protocol was developed a priori to guide the review process and ensure methodological consistency; however, it was not prospectively registered in a publicly accessible repository.

### 2.2. Review Question

The review question was developed according to the Population–Concept–Context (PCC) framework recommended by the Joanna Briggs Institute for scoping reviews [16].

The primary review question is: “What instruments, competency frameworks, questionnaires, screening tools, checklists, and other assessment approaches have been used to evaluate nutrition counselling in midwifery and perinatal care, and to what extent do existing assessment approaches capture women’s experiences of the quality of midwife-led perinatal nutrition counselling?”

To address the review question, the eligibility criteria were developed according to the Population–Concept–Context (PCC) framework recommended by the Joanna Briggs Institute. The PCC framework used to guide the review is presented in Table 2:

Population: Midwives, pregnant women, postpartum women, and healthcare professionals involved in nutrition counselling during the perinatal period.

Concept: Instruments, competency frameworks, questionnaires, screening tools, checklists, and other assessment approaches evaluating nutrition counselling, nutrition-related competencies, counselling quality, nutrition education, or women’s experiences of nutrition counselling.

Context: Antenatal, intrapartum, postpartum, and other perinatal healthcare settings, including hospital- and community-based maternity services, without geographical restrictions.

### 2.3. Eligibility Criteria

Eligibility criteria were established a priori according to the Population–Concept–Context (PCC) framework and the objectives of the review. Studies and documents were considered eligible if they described, developed, validated, applied, or evaluated instruments, competency frameworks, questionnaires, screening tools, checklists, or other assessment approaches related to nutrition counselling, nutrition education, nutrition-related competencies, counselling quality, or women’s experiences of nutrition counselling within midwifery or perinatal care.

Eligible sources included original quantitative, qualitative, and mixed-methods studies, methodological studies describing instrument development or validation, observational studies, consensus statements, clinical practice guidelines, competency frameworks, and reports from professional organizations. Publications involving midwives, pregnant women, postpartum women, or other healthcare professionals providing nutrition counselling during the perinatal period were considered eligible. Studies involving mixed professional samples, such as midwives and nurses, were eligible when midwives were explicitly represented in the study population and the assessment approach addressed nutrition counselling or nutrition-related care within antenatal, postnatal, or other perinatal settings. Such studies were retained because the purpose of the review was to map assessment approaches relevant to midwifery and perinatal nutrition care rather than to restrict the evidence base to instruments developed exclusively for midwives. Where professional groups were combined in the original study and midwife-specific findings could not be separated, the evidence was interpreted as provider-focused rather than as exclusively representative of midwifery practice.

For broad professional competency frameworks, eligibility additionally required explicitly identifiable nutrition- or nutrition-counselling-related competencies that could be extracted and mapped against the objectives of the review. General midwifery competency or professional assessment frameworks without sufficiently specific nutrition-related content were not considered eligible solely on the basis of their relevance to midwifery practice.

Studies were excluded if they focused exclusively on clinical nutritional interventions, dietary supplementation, or nutritional outcomes without evaluating any aspect of nutrition counselling or its assessment. Studies addressing general health education without a nutrition component, editorials, conference abstracts, letters, commentaries, protocols, and publications without sufficient methodological information were also excluded. Broader patient-experience, communication, shared decision-making, or person-centred care instruments were considered eligible only when they were applied to, or explicitly developed for, nutrition counselling within midwifery or perinatal care. Generic instruments assessing healthcare communication or patient experience without a specific nutrition-counselling component were excluded, as their inclusion would have extended beyond the predefined Concept and Context of the review. This criterion was applied intentionally to distinguish nutrition-specific assessment from the broader literature on general maternity-care experience and communication and to maintain alignment with the predefined review question.

No restrictions were applied regarding geographical setting or healthcare system in order to capture the broadest possible range of existing assessment approaches.

### 2.4. Information Sources

A comprehensive literature search was conducted in the following electronic bibliographic databases: PubMed/MEDLINE, Scopus, Web of Science Core Collection, CINAHL, and Embase. These databases were selected to ensure broad coverage of the biomedical, nursing, midwifery, public health, and allied health literature relevant to nutrition counselling and perinatal care. The search covered all eligible publications indexed from database inception to 1 July 2026, which served as the predefined final search date for the present review.

To maximize the identification of relevant evidence, the reference lists of all included studies and relevant review articles were manually screened for additional eligible publications. Grey literature was also considered through searches of the websites of major international organizations and professional bodies involved in maternal health, nutrition, and midwifery, including the World Health Organization (WHO), the International Confederation of Midwives (ICM), the International Federation of Gynecology and Obstetrics (FIGO), and the Nursing and Midwifery Board of Ireland (NMBI), where relevant competency frameworks, guidelines, or assessment tools may be available.

No restrictions regarding geographical setting were applied. Searches were limited to publications available in the English language because instrument development, competency frameworks, and methodological descriptions require accurate interpretation during data extraction and synthesis.

### 2.5. Search Strategy

The search strategy was developed in accordance with the recommendations of the Joanna Briggs Institute for scoping reviews and established recommendations for systematic literature searching [18], combining controlled vocabulary terms (e.g., Medical Subject Headings [MeSH], where applicable) with free-text keywords to maximize search sensitivity. Initially developed for PubMed/MEDLINE, it was subsequently adapted for each electronic database according to its specific indexing system and search interface. The strategy was refined iteratively through preliminary searches and examination of the terminology used in eligible studies, allowing additional search terms to be identified and incorporated to enhance the sensitivity and comprehensiveness of the review. Before the final searches were conducted, the electronic search strategy was independently reviewed by a second researcher for completeness, accuracy, and consistency; no formal structured peer-review framework, such as PRESS [19], was applied.

Search terms were selected to reflect the three core concepts of the review: (i) midwifery and maternity care, (ii) nutrition counselling and nutrition-related care, and (iii) assessment instruments, competency frameworks, questionnaires, checklists, and evaluation methods. Boolean operators (“AND”, “OR”), truncation symbols, and database-specific search functions were applied where appropriate to optimize retrieval.

Two complementary search strategies were used. The first focused on instruments, frameworks, questionnaires, checklists, and other assessment approaches related to nutrition counselling in midwifery and perinatal care. The second focused on counselling quality, patient-reported experience (PREM-related terminology), communication quality, person-centred care, shared decision-making, and related experience-based terminology, combined with nutrition- and maternity-related terms, to increase sensitivity for potentially relevant patient-reported or experience-based assessment approaches.

The complete database-specific electronic search strategies, including controlled vocabulary and free-text terms, Boolean operators, database-specific syntax, search coverage, and applied language restrictions, are provided in Supplementary Materials to enhance transparency and reproducibility. Across the electronic database searches, 186 records were identified before duplicate removal; database-specific retrieval counts were not retained and are therefore not reported separately.

### 2.6. Study Selection

All records identified through the electronic database searches were exported into reference management software, and duplicate records were removed prior to screening. The remaining records underwent a two-stage screening process. First, titles and abstracts were independently screened by two reviewers according to the predefined eligibility criteria. Studies considered potentially eligible subsequently underwent independent full-text assessment by the same two reviewers.

Disagreements regarding study eligibility were resolved through discussion and consensus between the two reviewers; consultation with a third reviewer was not required.

**Figure 1:**
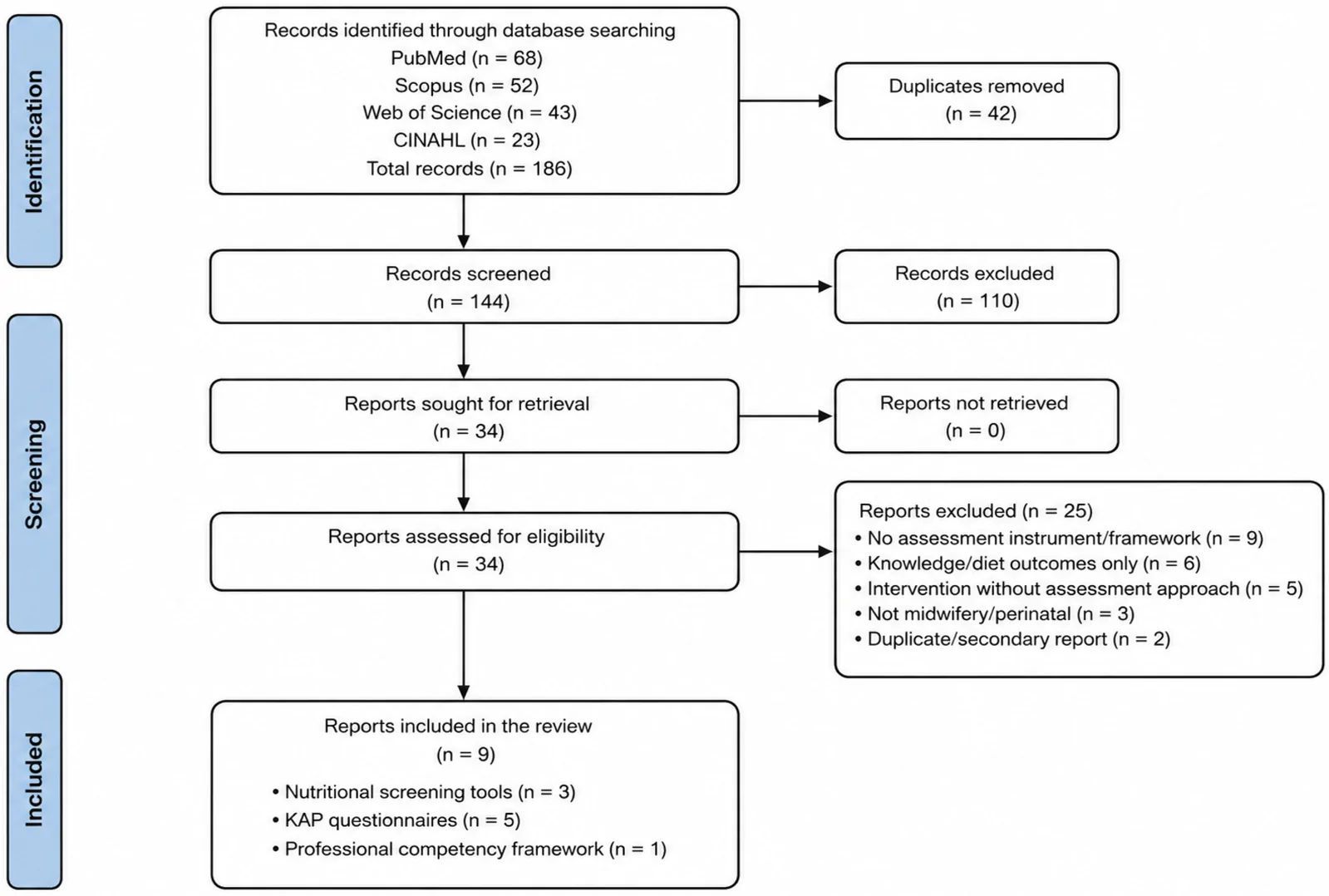
PRISMA-ScR flow diagram of the study selection process.

The study selection process is documented in a PRISMA-ScR flow diagram (Figure 1), reporting the numbers of records identified, screened, excluded, and included, together with the reasons for exclusion at the full-text screening stage, where applicable.

### 2.7. Data Charting

Data from the included sources were charted independently by two reviewers using a standardized data-charting form developed specifically for this review. The charting form was pilot-tested on a sample of eligible studies and refined, where necessary, to ensure consistency and completeness of data collection.

The following information was extracted, where applicable: author(s), year of publication, country, study design, study aim, healthcare setting, participant characteristics, instrument or assessment approach, target population, domains assessed, development methodology, validation procedures, psychometric properties (if reported), principal findings, and relevance to the objectives of the present review.

Any discrepancies in the charted data were resolved through discussion and consensus between the two reviewers; consultation with a third reviewer was not required.

The standardized data-charting form developed for this review is presented in Table 3. The form was designed to systematically capture the methodological characteristics, conceptual content, and measurement properties of existing assessment approaches while identifying potential gaps relevant to future patient-reported assessment of women’s experiences of midwife-led perinatal nutrition counselling.

Where available, information on instrument-development procedures and reported measurement properties, including content or face validation, pilot testing, reliability, validity, responsiveness, and other psychometric evidence, was extracted descriptively as reported in the original publications.

### 2.8. Data Synthesis

Data were synthesized descriptively using tabular and narrative approaches in accordance with the objectives of the scoping review. Extracted information was organized to map the characteristics of the identified assessment approaches, including their intended purpose, target population, domains assessed, development methodology, validation procedures, and reported psychometric properties.

The identified assessment approaches were grouped according to their primary purpose, such as competency frameworks, knowledge-, attitude-, and practice-based questionnaires, nutritional screening tools, observational assessment approaches, and patient-reported instruments. Similarities and differences across these categories were examined to identify the scope of existing assessment approaches and highlight areas of conceptual overlap or divergence.

Reported instrument-development, validation, and measurement-property evidence was also synthesized descriptively across approaches, with attention to the type and extent of validation procedures reported and to the availability of reliability, validity, responsiveness, and other psychometric information.

Particular emphasis was placed on identifying domains represented across the included assessment approaches and on distinguishing these from dimensions relevant specifically to women’s experience of the counselling process. The provisional conceptual synthesis was developed by integrating the domains identified through the formal evidence map with established principles of woman-centred maternity care and professional midwifery practice and with complementary qualitative evidence concerning women’s and midwives’ experiences of nutrition counselling. This synthesis was used to identify candidate domains potentially relevant to future patient-reported assessment; it was not intended to constitute a validated construct model or formal instrument-development framework.

Given the heterogeneity of the identified approaches in terms of purpose, target population, assessment format, measured constructs, and reported outcomes, quantitative pooling was neither appropriate nor consistent with the mapping objective of the present scoping review. The descriptive synthesis was therefore structured comparatively across assessment purpose, target respondent, domains assessed, mode of administration, and reported measurement characteristics, with particular attention to recurring domains, areas of conceptual overlap, and dimensions of counselling quality that were insufficiently represented across existing approaches.

### 2.9. Critical Appraisal

Consistent with current Joanna Briggs Institute (JBI) methodological guidance for scoping reviews, a formal critical appraisal of the included sources was not undertaken because the primary objective of this review was to identify, map, and characterize existing assessment approaches rather than evaluate the methodological quality or effectiveness of individual studies [16]. Similarly, no formal evaluation of measurement properties using the Consensus-based Standards for the selection of health Measurement Instruments (COSMIN) methodology [20] was undertaken. Instead, instrument-development procedures and reported validation and measurement-property evidence were mapped descriptively on the basis of information provided in the included publications. This included, where available, content or face validation, expert review, pre-testing or pilot testing, reliability, validity, responsiveness, and other reported psychometric evidence. Given the heterogeneity of the identified approaches in terms of purpose, respondent, format, construct, and stage of development, no comparative judgement or ranking of measurement quality across approaches was performed. This descriptive mapping was used to characterize the extent and nature of the available development and validation evidence and to identify methodological limitations and remaining measurement gaps relevant to the patient-reported assessment of women’s experiences of midwife-led perinatal nutrition counselling.

### 2.10. Presentation of Results

The results of the review are presented using descriptive tables, figures, and narrative synthesis in accordance with the objectives of the review. Identified assessment approaches were categorized according to their primary purpose, target population, methodological characteristics, domains assessed, instrument-development procedures, validation evidence, and reported measurement properties. Particular attention was given to identifying conceptual similarities, methodological differences, and areas insufficiently addressed by existing assessment approaches.

Summary tables were developed to compare the characteristics and intended purposes of the included assessment approaches and to map the nutrition- and counselling-related domains represented across them. Reported development, validation, and measurement-property evidence was additionally summarized across approaches to illustrate the extent and nature of the available evidence without implying comparative psychometric quality. The findings were synthesized to identify current measurement gaps and to generate a provisional conceptual mapping of domains relevant to the patient-reported assessment of women’s experiences of midwife-led perinatal nutrition counselling.

## 3. Results

### 3.1. Search Results

The database search identified 186 records. Following the removal of 42 duplicate records, 144 unique citations remained for title and abstract screening. After the initial screening, 34 articles were retrieved for full-text assessment. Twenty-five studies were subsequently excluded because they did not evaluate an assessment approach related to nutrition counselling in midwifery or perinatal care, focused exclusively on nutritional knowledge or dietary outcomes, or lacked an identifiable assessment instrument or framework. The relatively small number of included assessment approaches reflects the deliberately nutrition-specific eligibility criteria of the review rather than the breadth of the wider maternity-care, communication, or patient-experience literature. To be retained, an assessment approach was required to include an explicit component addressing nutrition counselling or nutrition-related care within a midwifery or perinatal context. Broader patient-reported, communication, or person-centred maternity-care instruments without a distinct nutrition-specific component were therefore outside the predefined Concept of the review and were not included in the final mapping.

Ultimately, nine reports met the eligibility criteria and were included in this scoping review. These reports represented distinct categories of evidence, including nutritional screening tools, knowledge-, attitude-, and practice-based questionnaires, structured observational approaches, and professional competency frameworks. Importantly, the nine included reports did not correspond to nine unique instruments or frameworks, as multiple publications evaluated or applied the same underlying assessment tool, particularly the FIGO Nutrition Checklist. The complete study selection process, including identification, duplicate removal, screening, full-text assessment, exclusions, and final inclusion, is presented in the PRISMA-ScR flow diagram (Figure 1).

### 3.2. Overview of the Identified Assessment Approaches

The nine included reports represented a broad spectrum of methods used to evaluate nutrition-related aspects of midwifery and perinatal care; however, they represented a smaller number of distinct assessment approaches because three reports evaluated or applied the same underlying instrument, the FIGO Nutrition Checklist. For synthesis, the distinct assessment approaches were organized according to their intended purpose and respondent into nutritional-risk screening tools, provider-focused knowledge-, attitude-, and practice-based (KAP) instruments, observational approaches to counselling delivery, and professional competency frameworks; patient-reported experience measures were considered as a distinct category but were not identified among the eligible approaches. These categories were not treated as conceptually or psychometrically equivalent; rather, each was interpreted according to its intended construct and purpose within nutrition-related maternity care. None of the identified assessment approaches was specifically designed to comprehensively evaluate women’s experiences of nutrition counselling delivered by midwives during the perinatal period. In practical terms, these approaches provide different types of information: nutritional-risk screening tools identify nutrition-related concerns in women; provider-focused questionnaires assess professionals’ knowledge, attitudes, confidence, and self-reported practices; observational approaches document aspects of counselling actually delivered during clinical encounters; and competency frameworks define the professional knowledge, skills, and behaviours expected of midwives. Patient-reported experience measures, had eligible nutrition-specific examples been identified, would instead provide information on how women themselves experienced the counselling process. The main characteristics of the identified assessment approaches are presented in Table 4.

### 3.3. Nutritional Screening Tools

Three included reports concerned nutritional screening and were based on the same underlying instrument, the FIGO Nutrition Checklist. These reports addressed different aspects of its development, acceptability, implementation, or clinical application.

The FIGO Nutrition Checklist, described by Killeen et al. [4], is an evidence-based clinical screening instrument intended to assist healthcare professionals in identifying dietary risk factors, promoting nutrition awareness, and facilitating individualized nutrition discussions during routine antenatal consultations. Rather than serving as a diagnostic or outcome assessment instrument, the checklist functions as a structured screening tool to support early identification of nutritional concerns and appropriate referral when indicated. Jacob et al. (2022) [21] evaluated the acceptability and feasibility of implementing the FIGO Nutrition Checklist across different clinical settings. Their assessment focused on healthcare professionals’ and pregnant women’s perceptions regarding the usability, practicality, and integration of the checklist into routine maternity care. The study examined implementation-related outcomes rather than clinical effectiveness or the quality of nutrition counselling. Grammatikopoulou et al. (2023) [22] applied the FIGO Nutrition Checklist in a cohort of pregnant women to evaluate dietary quality and nutrition-related risk. The assessment focused on dietary behaviours, adherence to nutritional recommendations, and the identification of women requiring additional nutritional support. The checklist was used as a nutritional screening instrument within routine antenatal care rather than as a measure of counselling quality or women’s experiences.

Accordingly, the FIGO Nutrition Checklist was interpreted in this review according to its intended function as a nutritional-risk screening and discussion-facilitation tool, rather than as a measure of counselling experience.

### 3.4. Provider-Focused and Observational Assessment Approaches

Five included reports examined healthcare professionals’ nutrition knowledge, attitudes, practices, or counselling delivery, although they differed in methodological design and mode of assessment. Most used questionnaire-based methods to assess healthcare professionals’ nutrition knowledge, confidence, attitudes, counselling practices, educational needs, and implementation of nutrition care, whereas one study incorporated qualitative interviews and structured observation of counselling delivery.

For synthesis, questionnaire-based approaches were interpreted primarily as measures of provider knowledge, attitudes, confidence, and self-reported practice, whereas observational evidence was interpreted as reflecting aspects of counselling delivery and implementation. These constructs were considered separately and were not treated as interchangeable indicators of counselling quality.

In contrast to the questionnaire-based approaches, Nankumbi et al. [9] used qualitative interviews and structured observations of group and one-to-one nutrition education to examine nutrition-related counselling practices in antenatal care. This approach provided direct information on counselling delivery rather than relying exclusively on provider self-report.

Kumbiley et al. (2021) [10] used a structured questionnaire to investigate nutrition care practices among midwives and nurses providing antenatal and postnatal care. The assessment focused on the frequency of nutrition-related activities, together with the barriers, facilitators, and determinants influencing the provision of nutrition care in clinical practice.

Masesane et al. (2024) [11] employed a combined assessment approach consisting of a structured interviewer-administered questionnaire and a standardized observational checklist. This approach evaluated midwives’ nutrition knowledge, counselling practices, and the integration of nutrition-related activities into routine antenatal care, while the observational component documented the implementation of nutrition counselling during clinical encounters.

Arrish et al. (2016) [23] conducted a cross-sectional survey examining Australian midwives’ nutrition knowledge, attitudes, confidence, and educational practices during pregnancy. The questionnaire explored self-reported preparedness to provide nutrition education and identified areas requiring further professional development.

Olloqui-Mundet et al. (2024) [24] administered a cross-sectional questionnaire to examine nutrition education practices among midwives in Spain. The survey assessed the delivery of nutrition education during routine maternity care, perceived barriers to counselling, previous nutrition training, and continuing educational needs related to maternal nutrition.

### 3.5. Professional Competency Framework

One professional competency framework met the eligibility criteria of this review. The International Confederation of Midwives (ICM) Essential Competencies for Midwifery Practice (2024) [5] provides an internationally recognized framework describing the core competencies expected of midwives across the continuum of reproductive, maternal, newborn, and postnatal care. The framework encompasses knowledge, clinical skills, professional behaviours, health promotion, communication, and evidence-informed practice, including competencies related to maternal nutrition and the provision of nutrition education. Rather than functioning as a questionnaire or screening instrument, the ICM framework serves as a competency-based reference for midwifery education, professional regulation, curriculum development, and workforce assessment.

The WHO Regional Competency Assessment Tool and the NMBI Competence Assessment Tool for Midwives, which had also been identified as potentially relevant professional competency resources, were reassessed against the same eligibility criteria. Although both were relevant to the broader assessment of midwifery competence, they were not included in the formal evidence map because they did not provide sufficiently specific, extractable nutrition-counselling content aligned with the nutrition-specific scope of the present review. Their exclusion was therefore based on the construct and scope assessed rather than on whether they captured women’s experiences of counselling.

Collectively, the identified assessment approaches reflected substantial variation in their intended purpose, target population, and assessment domains, ranging from nutritional screening and professional competency assessment to the evaluation of nutrition knowledge, attitudes, confidence, and counselling practices.

### 3.6. Reported Development, Validation, and Measurement Properties

The extent and nature of instrument-development and validation evidence varied substantially across the identified assessment approaches (Table 5). Several questionnaire-based approaches reported preliminary development procedures, including adaptation of items from previous studies, expert assessment of content or face validity, and pre-testing or pilot testing. For example, the questionnaire used by Kumbiley et al. underwent expert assessment of content validity and pre-testing, while Masesane et al. reported content and face validation by relevant professionals together with pilot testing. Olloqui-Mundet et al. described a structured expert-judgement process evaluating item sufficiency, clarity, coherence, and relevance, followed by questionnaire refinement and pilot testing.

However, formal measurement-property evidence was limited and inconsistently reported across the included approaches. Quantitative reliability coefficients, evidence of construct or criterion validity, measurement error, responsiveness, and other psychometric properties relevant to standardized measurement instruments were generally unavailable or were not comprehensively evaluated within the included publications. Moreover, several included approaches were not psychometric instruments by design: the FIGO Nutrition Checklist studies primarily examined nutritional screening, acceptability, feasibility, or clinical application; the Nankumbi study used qualitative interviews and structured observation to characterize counselling delivery; and the ICM Essential Competencies constitute a professional competency framework rather than a patient-reported measurement instrument.

Given these substantial differences in purpose, respondent, format, construct, and stage of instrument development, direct comparison or ranking of psychometric performance across approaches was neither methodologically appropriate nor supported by the available evidence. The present review therefore maps the development and validation evidence reported for each approach descriptively, without inferring comparative measurement quality.

### 3.7. Cross-Cutting Synthesis of Assessment Domains and Measurement Gaps

Cross-cutting comparison of the identified approaches revealed four conceptually distinct measurement levels: (1) nutritional risk and dietary status, (2) professional knowledge and competence, (3) counselling delivery and implementation, and (4) women’s experience of nutrition counselling. The first three levels were represented to varying degrees across the included approaches. The extent of development and validation evidence also varied across these levels, with some questionnaire-based approaches reporting content or face validation and pre-testing or pilot testing, while formal measurement-property evidence remained limited and several approaches were not psychometric instruments by design. Nutritional screening tools primarily addressed dietary risk and the identification of nutrition-related concerns; KAP questionnaires predominantly assessed providers’ knowledge, confidence, attitudes, self-reported practices, and educational needs; observational approaches captured aspects of whether and how nutrition-related activities were delivered in clinical encounters; and competency frameworks defined expected professional knowledge, skills, and behaviours. No eligible nutrition-specific patient-reported experience measure was identified to represent the fourth level, women’s experience of the counselling process.

**Figure 2:**
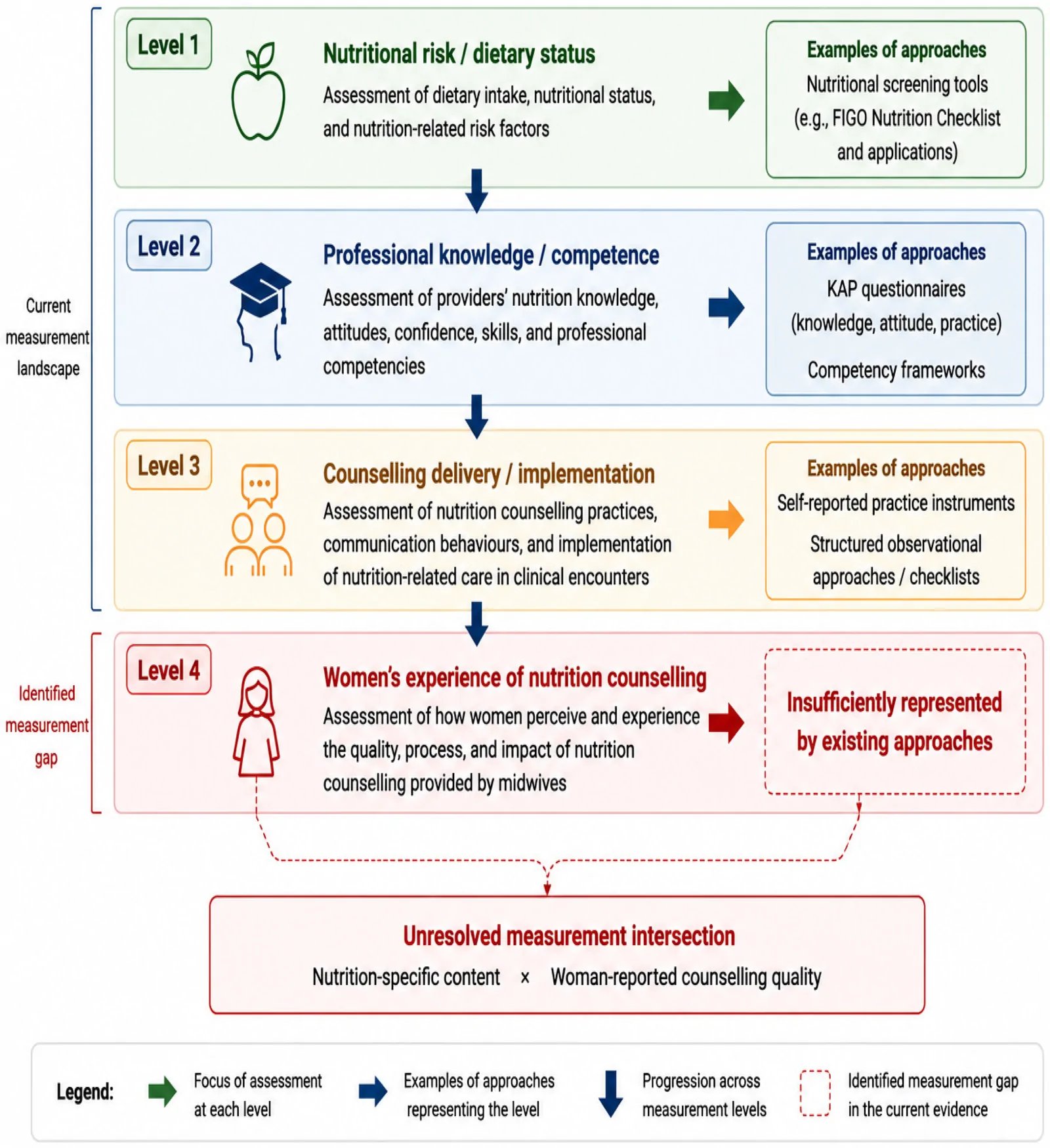
Conceptual mapping of existing assessment approaches and the identified measurement gap in evaluating women’s experiences of midwife-led nutrition counselling during the perinatal period.

Across these categories, several areas of conceptual convergence were evident. Communication, provision of nutrition information, professional preparedness, counselling activity, and support for nutrition-related decision-making appeared across more than one type of assessment approach, although they were operationalized from different perspectives. At the same time, important areas of divergence were identified. Existing approaches differed in whether they assessed the recipient’s nutritional risk, the provider’s competence or behaviour, or the implementation of counselling activities, and these constructs were generally measured independently rather than as components of an integrated counselling-quality construct. To illustrate this cross-cutting synthesis, Figure 2 maps the identified assessment approaches across four conceptually distinct measurement levels and highlights the area that remains insufficiently represented. As shown in the figure, the identified evidence was distributed across distinct measurement purposes, predominantly addressing nutritional risk and dietary status, professional knowledge and competence, and counselling delivery or implementation. No eligible nutrition-specific patient-reported experience measure was identified, leaving women’s reported experience of the nutrition-counselling process unrepresented as a distinct measurement purpose within the included evidence.

This mapping therefore identifies a structural measurement gap rather than simply the absence of a single comprehensive instrument. The identified approaches predominantly assessed what nutritional risks were present, what healthcare professionals knew or reported doing, and whether nutrition-related activities were implemented. They provided substantially less information on how women perceived and experienced the counselling process itself. Accordingly, the principal gap lies at the intersection between nutrition-specific content and woman-reported counselling quality, rather than in the complete absence of nutrition-related assessment tools. This distinction provides a more specific basis for subsequent work to determine whether relevant domains can be captured through adaptation or combination of existing measures or whether development of a new patient-reported instrument is justified.

## 4. Discussion

This scoping review mapped the currently available assessment approaches related to nutrition counselling in midwifery and perinatal care. The findings revealed substantial methodological diversity, with the identified approaches encompassing nutritional screening tools, knowledge-, attitude-, and practice-based (KAP) questionnaires, structured observational approaches, and professional competency frameworks. These approaches differed considerably in their intended purpose, target population, and assessment constructs, reflecting the multidimensional nature of nutrition-related care within midwifery practice. Collectively, the findings provide an overview of the current landscape of assessment approaches and establish a foundation for examining their scope, strengths, and remaining areas requiring further methodological development. Where relevant, additional literature not included among the sources formally mapped in this scoping review is cited in the Discussion solely to contextualize and interpret the findings; these references were not treated as included evidence and did not contribute to the descriptive mapping or synthesis.

The first group of identified assessment approaches focused on nutritional screening rather than on the evaluation of nutrition counselling itself. The FIGO Nutrition Checklist, described by Killeen et al. [4], represents one of the most widely adopted structured approaches for identifying nutritional risk factors during pregnancy and facilitating nutrition-related discussions within routine antenatal care. Beyond the evidence formally mapped in the present review, subsequent studies have demonstrated the feasibility and acceptability of the FIGO Nutrition Checklist across different healthcare settings, supporting its integration into routine maternity practice (Jacob et al. [21]; Grammatikopoulou et al. [22]). Additional recent literature has further illustrated the clinical value of structured nutritional assessment, suggesting that systematic screening may facilitate the identification of women at increased nutritional risk and support timely dietary guidance within antenatal services (Wahabi et al. [25]; Fayed et al. [26]). Collectively, these findings indicate that contemporary maternity care increasingly recognizes the importance of standardized nutritional screening as an integral component of antenatal practice. Nevertheless, these approaches primarily support nutritional risk identification and clinical decision-making rather than evaluation of the counselling experience from the woman’s perspective.

Although the identified provider-focused assessment approaches differed in methodological design and clinical context, they consistently examined nutrition-related care from the perspective of healthcare professionals or through observation of professional practice rather than from the perspective of women receiving care. Across diverse healthcare settings, questionnaire-based and observational approaches examined midwives’ or nurses’ nutrition knowledge, confidence, attitudes, counselling practices, educational needs, perceived barriers, and aspects of counselling delivery (Nankumbi et al. [9]; Kumbiley et al. [10]; Masesane et al. [11]; Arrish et al. [23]; Olloqui-Mundet et al. [24]). This consistent focus reflects the longstanding recognition that healthcare professionals’ nutritional competence is a key determinant of the quality of nutrition care provided during pregnancy. Contextual evidence beyond the studies formally mapped in this review has similarly shown that midwives recognize the importance of nutrition counselling while reporting limited education, insufficient training, lack of confidence, and time constraints as barriers to its effective implementation (Arrish et al. [27]; Rizk et al. [28]). Related literature has also highlighted the strengthening of nutrition education within midwifery practice as an important strategy for improving the consistency and quality of antenatal nutrition counselling (Olloqui-Mundet et al. [29]). Taken together, these findings demonstrate that current provider-focused assessment approaches predominantly address professional preparedness, practices, and aspects of counselling delivery. These provider-focused constructs are conceptually distinct from women’s reported experience of the counselling encounter.

The findings of this review further indicate that nutrition-related assessment within midwifery extends beyond questionnaires and screening instruments to include professional competency frameworks that define the expected standards of clinical practice. The International Confederation of Midwives (ICM) Essential Competencies for Midwifery Practice establishes nutrition education and health promotion as core components of midwifery care throughout the reproductive continuum, emphasizing the responsibility of midwives to provide evidence-informed nutritional guidance and support (ICM, 2024) [5]. This interpretation is further supported by recent literature discussing the revised ICM competencies, which reinforces the recognition of nutrition as an element of contemporary midwifery practice and the broader international emphasis on the knowledge, skills, and professional behaviours expected from the profession (Embo et al. [30]). Similarly, regional and national competency assessment frameworks, including those developed by the World Health Organization [6] and the Nursing and Midwifery Board of Ireland [7], emphasize the evaluation of professional competence, clinical performance, and educational preparedness. Among these broader professional competency resources, only the ICM framework met the nutrition-specific eligibility criteria of the present review. The WHO and NMBI tools were retained as contextual examples of broader midwifery competency assessment but were not included in the formal evidence map because sufficiently specific nutrition-counselling content could not be extracted for the purposes of this review. Collectively, these frameworks provide robust standards for assessing professional capability and supporting workforce development; however, they are principally designed to evaluate the competencies of healthcare professionals rather than the experiences or perceptions of women receiving nutrition counselling.

Beyond the assessment approaches formally mapped in this review, an emerging body of qualitative research provides complementary contextual evidence regarding nutrition counselling from the perspective of pregnant women. Qualitative evidence demonstrates that women value nutrition counselling that is individualized, practical, continuous, and responsive to their personal circumstances rather than limited to the provision of standardized dietary information (Lorenz et al. [31]; van Lonkhuijzen et al. [32]). Women consistently reported greater engagement when counselling was delivered within supportive therapeutic relationships, encouraged shared decision-making, and addressed their individual concerns and everyday challenges. Similarly, midwives have acknowledged the potential benefits of personalized nutrition counselling while identifying organizational barriers, limited consultation time, insufficient nutrition training, and restricted access to supportive digital resources as factors influencing counselling delivery (de Wit et al. [33]; van Lonkhuijzen et al. [34]). These findings suggest that the perceived quality of nutrition counselling is shaped not only by healthcare professionals’ knowledge and competencies but also by the interpersonal characteristics of the counselling encounter and the extent to which care is experienced as relevant, supportive, and responsive to women’s needs.

Within the context of the present review, high-quality midwife-led perinatal nutrition counselling from the woman’s perspective was therefore conceptualized as an evidence-informed, woman-centred process in which nutrition-related information and support are communicated clearly, individualized and practically applicable, responsive to women’s clinical, cultural, and personal circumstances, supportive of shared decision-making, continuous where appropriate, and delivered within a supportive and non-judgmental therapeutic relationship. This working construct was informed by the domains mapped across the included nutrition-related assessment approaches, established principles of woman-centred maternity care and professional midwifery practice, and complementary qualitative evidence concerning women’s and midwives’ experiences of nutrition counselling [5,31,32,33,34]. It should not be interpreted as a formally validated definition or measurement model, but as an explicit conceptual framework for the provisional synthesis undertaken in this review.

Applying this working construct to the cross-cutting synthesis, seven provisional candidate domains were identified for consideration in future patient-reported assessment: (1) clarity and comprehensibility of nutrition-related communication; (2) individualization and practical applicability of dietary guidance; (3) responsiveness to women’s clinical, cultural, and personal circumstances; (4) involvement in nutrition-related decision-making; (5) continuity and consistency of counselling support; (6) the supportive and non-judgmental nature of the counselling relationship; and (7) women’s perceived support for understanding and acting on nutrition-related recommendations. These domains should not be interpreted as a definitive measurement model or as predetermined questionnaire subscales. Rather, they represent a review-derived conceptual starting point that requires further refinement through qualitative work with women and relevant stakeholders, assessment of content validity, and subsequent psychometric evaluation before any instrument structure can be established. The seven provisional candidate domains and the evidence informing their identification are summarized in Table 6.

These domains represent a provisional review-derived conceptual synthesis and should not be interpreted as validated constructs, predetermined questionnaire subscales, or a finalized measurement model. Further qualitative exploration, stakeholder involvement, content-validation procedures, and psychometric evaluation are required before instrument structure can be established.

The present findings also highlight an important methodological distinction between existing nutrition-related assessment instruments and the construct addressed by the present review. The present synthesis further showed that the extent of instrument-development and validation evidence was uneven across the identified approaches: some questionnaire-based approaches incorporated expert content or face validation and pre-testing or pilot testing, whereas comprehensive evaluation of measurement properties was generally limited, and several identified approaches were not psychometric instruments by design. Recent literature beyond the sources formally mapped in this review has described the development of psychometrically evaluated instruments addressing specific nutrition-related domains, including maternal nutrition knowledge (Callegaro et al. [35]), nutrition and food safety knowledge during pregnancy (Zahra et al. [36]), and the implementation of dietary interventions or nutritional recommendations within antenatal care (Beulen et al. [37]; De Vito et al. [38]). These developments reflect increasing methodological sophistication and demonstrate growing recognition of the importance of nutritional assessment during pregnancy. However, each of these instruments was developed to evaluate discrete constructs, such as knowledge acquisition, food safety awareness, guideline implementation, or intervention delivery, rather than the quality of nutrition counselling as experienced by pregnant women. However, none of the approaches identified in the present review constituted a validated patient-reported instrument capable of comprehensively evaluating women’s experiences of midwife-led nutrition counselling throughout the perinatal period.

The fragmentation observed across the identified assessment approaches may partly reflect the way nutrition-related care has historically been conceptualized and evaluated within maternity services. Measurement has largely developed around distinct clinical and professional objectives, including the identification of nutritional risk, assessment of healthcare professionals’ knowledge and competence, and documentation of counselling activities, rather than around nutrition counselling as an integrated care experience from the woman’s perspective. This separation of assessment purposes may help explain why communication, individualization, shared decision-making, continuity, and behavioural support appear across the literature but are rarely operationalized together within a nutrition-specific patient-reported construct. The resulting measurement gap may therefore reflect not simply the absence of an instrument, but a broader conceptual separation between what is clinically assessed, what professionals are expected to know or deliver, and how women experience the counselling they receive.

The limited representation of nutrition-specific patient-reported assessment within the approaches identified in this review has important implications for both clinical practice and research. Without an assessment approach capable of comprehensively capturing women’s experiences of nutrition counselling, healthcare providers and researchers have limited opportunities to systematically assess the quality, consistency, and perceived effectiveness of nutrition counselling delivered throughout the perinatal period. Consequently, the multidimensional aspects of woman-centred nutrition counselling identified through the present synthesis remain difficult to evaluate comprehensively using existing assessment approaches. In this context, the measurement gap identified by the present review is most appropriately framed as a gap in patient-reported experience measurement (PREM), rather than patient-reported outcome measurement (PROM). A future PREM would assess women’s perceptions of how nutrition counselling was delivered and experienced; changes in nutrition knowledge, self-efficacy, dietary behaviour, or clinical outcomes would constitute separate outcome constructs and would require distinct assessment. As maternity care continues to adopt more personalized and evidence-informed models of care, the availability of valid and reliable patient-reported experience measures may facilitate quality improvement initiatives, support service evaluation, identify unmet counselling needs, and contribute to the development of more responsive nutrition counselling strategies within routine midwifery practice.

### 4.1. Strengths and Limitations

This review has several strengths. To the best of our knowledge, this is the first scoping review to systematically map assessment approaches specifically related to nutrition counselling within midwifery and perinatal care. By examining nutritional screening tools, competency frameworks, knowledge-, attitude-, and practice-based questionnaires, and structured observational approaches within a single conceptual framework, the review provides a comprehensive overview of how nutrition-related care is currently assessed across different dimensions of maternity services. Furthermore, the inclusion of both empirical studies and internationally recognized professional frameworks enabled a broader understanding of existing assessment strategies and facilitated the identification of methodological differences among available approaches.

Several limitations should also be acknowledged. First, although a comprehensive search strategy was employed, relevant assessment approaches published in languages other than English or reported in grey literature may not have been identified. Second, the review focused on mapping existing assessment approaches rather than formally appraising their psychometric performance or measurement quality. Instrument-development procedures and reported measurement properties were therefore mapped descriptively rather than formally evaluated using a framework such as COSMIN [20]. Although several included approaches reported content or face validation, expert review, pre-testing, or pilot testing, comprehensive psychometric evaluation was inconsistent and generally limited. Moreover, the heterogeneity of the identified approaches in terms of purpose, respondent, format, construct, and stage of development precluded meaningful direct comparison of their measurement properties. The present review therefore describes the available development and validation evidence but does not provide a comparative judgement or ranking of measurement quality across approaches. In addition, some included provider-focused studies involved mixed professional samples, including both midwives and nurses, and did not always report findings separately by professional group. Such evidence was therefore interpreted as relevant to the broader provider-focused assessment landscape rather than as exclusively representative of midwifery practice. Finally, the considerable heterogeneity in study designs, target populations, assessment objectives, and outcome measures limited direct comparisons across studies and precluded quantitative synthesis.

### 4.2. Future Research

The findings of this review suggest several priorities for future research. Beyond the continued development of evidence-based nutritional interventions during pregnancy, future research should build on the candidate domains identified through the present synthesis and further examine how women’s experiences of nutrition counselling can be appropriately assessed across the continuum of midwifery care. Qualitative enquiry and formal content-validation procedures involving women and relevant stakeholders are needed to refine these candidate domains and to determine whether they can be adequately represented through the adaptation or combination of existing measures or whether the development of a new patient-reported experience measure (PREM) is warranted. Any subsequent instrument-development or adaptation process should include rigorous evaluation of measurement properties and cross-cultural validity using appropriate methodological standards. Such work could facilitate meaningful comparisons across healthcare settings, strengthen quality-assurance initiatives, and contribute to the delivery of more responsive and evidence-informed nutrition counselling within routine maternity services.

Overall, this review demonstrates that considerable progress has been achieved in the development of assessment approaches addressing nutritional risk, professional competencies, nutrition knowledge, and counselling practices within maternity care. Nevertheless, the existing evidence remains fragmented across distinct assessment domains, with the intersection between nutrition-specific content and woman-reported counselling quality remaining insufficiently represented. Advancing the evaluation of nutrition counselling from a woman-centred perspective may strengthen both clinical practice and research by enabling more comprehensive assessment of counselling quality and supporting the continuous improvement in nutrition care throughout the perinatal period.

## 5. Conclusions

This scoping review systematically mapped the currently available assessment approaches related to nutrition counselling in midwifery and perinatal care. Nine reports met the eligibility criteria, although these did not represent nine unique assessment approaches, as three reports evaluated or applied the FIGO Nutrition Checklist. The included evidence comprised nutritional-risk screening tools, provider-focused knowledge-, attitude-, and practice-based questionnaires, observational approaches to counselling delivery, and a professional competency framework, each addressing a distinct measurement purpose. Development and validation evidence varied substantially across these approaches: some questionnaire-based assessments reported content or face validation and pre-testing or pilot testing, whereas comprehensive psychometric evaluation was generally limited, and conventional psychometric properties were not applicable to observational approaches or competency frameworks by design. No eligible nutrition-specific patient-reported experience measure was identified for assessing women’s experiences of midwife-led nutrition counselling throughout the perinatal period. These findings identify an important methodological gap and support further research to determine whether existing measures can be adapted or combined to address this gap or whether the development and psychometric evaluation of a new patient-reported experience measure (PREM) is warranted.

This scoping review represents an initial step within a broader, sequential programme of research on the patient-reported assessment of midwife-led perinatal nutrition counselling. The identified gap in nutrition-specific patient-reported experience measurement and the provisional domains emerging from the synthesis will inform subsequent methodological phases aimed at refining the construct with women and relevant stakeholders and determining whether existing measures can adequately capture these domains. If development of a new patient-reported experience measure (PREM) is supported by these subsequent phases, stakeholder-informed item development, content validation, and rigorous psychometric evaluation will be required before its use in research or clinical practice.

## Figures and Tables

**Table 1:** Characteristics, scope, and limitations of existing approaches used to evaluate nutrition-related care in midwifery practice.

Category	Representative Examples	Primary Purpose	Respondent/Assessor	What Is Evaluated	Interpretation According to Intended Purpose
Professional competency frameworks	ICM Essential Competencies for Midwifery Practice; broader contextual examples include the WHO Regional Competency Assessment Tool and NMBI Competence Assessment Tool	Define expected competencies for nutrition-related care	Midwives/educators/assessors	Professional knowledge, skills, health promotion competencies	Designed to define and assess professional competencies rather than patient-reported counselling experience
Knowledge, confidence and practice questionnaires (KAP)	Nankumbi et al.; Kumbiley et al.; Botswana study	Assess nutrition knowledge, confidence, self-reported practice and barriers	Midwives	Nutrition knowledge, confidence, attitudes, self-reported counselling practices	Designed primarily to assess provider knowledge, confidence, attitudes, practices, and perceived barriers
Nutritional screening tools	FIGO Nutrition Checklist	Identify nutritional risks and facilitate nutrition discussions	Pregnant woman with healthcare professional	Nutritional risk factors and dietary behaviours	Designed to identify nutritional risk and facilitate nutrition-related discussion
Structured observational approaches	Botswana observational checklist	Assess whether predefined nutrition counselling components are delivered during consultations	Independent observer	Delivery of predefined counselling components	Designed to document observable counselling delivery and implementation

**Table 2:** Population–Concept–Context (PCC) eligibility framework used to guide the scoping review.

PCC Element	Description
Population	Midwives, pregnant women, postpartum women, and other healthcare professionals involved in perinatal nutrition counselling
Concept	Assessment of nutrition counselling, nutrition competencies, counselling quality, nutrition education, patient experience, nutrition-related practice
Context	Antenatal, intrapartum, postpartum and community maternity care settings

**Table 3:** Standardized data-charting form used for data-charting form the included sources.

Data Item	Description/Information Extracted
Citation	First author, year of publication
Country	Country where the study or instrument was developed and/or applied
Study design	Quantitative, qualitative, mixed-methods, methodological study, guideline, competency framework, consensus study, observational study
Study objective	Primary aim of the study
Healthcare setting	Antenatal care, maternity hospital, primary care, community care, postpartum care, or other
Target population	Midwives, pregnant women, postpartum women, educators, or other healthcare professionals
Instrument/assessment approach	Name of the questionnaire, framework, checklist, screening tool, or assessment method
Instrument category	Competency framework, KAP questionnaire, nutritional screening tool, observational checklist, patient-reported questionnaire, or other
Intended respondent/assessor	Midwife, pregnant woman, observer, educator, assessor, or healthcare professional
Purpose of the instrument	Assessment of nutrition knowledge, competencies, counselling practices, counselling quality, nutritional risk, patient experience, or other
Domains assessed	Nutrition knowledge, communication, personalization, counselling practices, behaviour change, nutritional risk, referral, documentation, follow-up, or other domains evaluated
Instrument development process	Literature review, expert panel, Delphi consensus, adaptation of an existing instrument, de novo development, or not reported
Validation procedures	Content validity, construct validity, criterion validity, reliability testing, pilot testing, psychometric evaluation, or not reported
Psychometric properties	Cronbach’s α, intraclass correlation coefficient (ICC), factor analysis, content validity index (CVI), measurement error, or other reported psychometric properties
Main findings	Principal findings relevant to nutrition counselling assessment
Conceptual dimensions not addressed	Nutrition- or counselling-related domains not addressed by the assessment approach, recorded descriptively where relevant to its intended purpose
Relevance to the present review	Contribution to identifying measurement gaps and informing future patient-reported assessment of women’s experiences of midwife-led perinatal nutrition counselling.

**Table 4:** Characteristics of the included reports and underlying assessment approaches related to nutrition counselling in midwifery and perinatal care.

Study/Report	Country/Scope	Target Participants	Assessment Approach	Instrument/Approach Type	Main Constructs Assessed	Development/Evaluation	Intended Measurement Purpose
Killeen et al., 2023 [4]	International	Pregnant women and healthcare professionals	FIGO Nutrition Checklist	Nutritional screening and counselling-support tool	Dietary risk identification; nutrition-related discussion	Evidence-based FIGO checklist discussed and applied as a clinical counselling-support resource	Identification of nutritional risk and facilitation of nutrition-related discussion
Jacob et al., 2022 [21]	Multinational	Women and healthcare practitioners	FIGO Nutrition Checklist	Nutritional screening tool—acceptability/feasibility evaluation	Acceptability; feasibility; implementation	Evaluation of the existing FIGO Nutrition Checklist in preconception and early-pregnancy care	Evaluation of acceptability and feasibility of the FIGO Nutrition Checklist
Grammatikopoulou et al., 2023 [22]	Greece	Pregnant women	FIGO Nutrition Checklist	Nutritional screening tool	Dietary quality; nutritional risk; dietary behaviours	Clinical application of the existing FIGO Nutrition Checklist	Assessment of dietary quality, dietary behaviours, and nutritional risk using the FIGO Nutrition Checklist
Nankumbi et al., 2020 [9]	Uganda	Midwives	Qualitative interviews and structured observation of nutrition education	Qualitative and observational assessment approach	Nutrition-education practices; counselling delivery; implementation of nutrition-related care	Qualitative interviews and structured non-participant observation; not a standardized psychometric instrument	Assessment of nutrition-education and counselling delivery in antenatal care
Kumbiley et al., 2021 [10]	Ghana	Midwives and nurses	Nutrition-care practice questionnaire	Provider-focused questionnaire	Nutrition-care practices; barriers; facilitators; determinants	Items adapted from previous studies and supplemented by author-developed items; content validation and pre-testing reported	Assessment of provider nutrition-care practices and determinants of care delivery
Masesane et al., 2024 [11]	Botswana	Midwives	Questionnaire with direct observation	Provider-focused questionnaire and observational checklist	Nutrition knowledge; counselling practices; observed clinical practice	Adapted questionnaire; expert content/face validation and pilot testing reported	Assessment of provider nutrition knowledge and counselling practices, including observed implementation
Arrish et al., 2016 [23]	Australia	Midwives	Nutrition knowledge, attitudes, and confidence survey	Provider-focused KAP questionnaire	Nutrition knowledge; attitudes; confidence; educational practices	Cross-sectional web-based questionnaire	Assessment of provider nutrition knowledge, attitudes, confidence, and educational practices
Olloqui-Mundet et al., 2024 [24]	Spain	Midwives	Nutrition-education practice survey	Provider-focused cross-sectional questionnaire	Nutrition education; counselling practices; barriers; training needs	Questionnaire based on previous work; expert-judgement content assessment and pilot testing reported	Assessment of provider nutrition-education practices, barriers, and training needs
International Confederation of Midwives (ICM), 2024 [5]	International	Midwives	Essential Competencies for Midwifery Practice	Professional competency framework	Professional knowledge; skills; behaviours; nutrition-related competencies	International professional competency framework defining standards for midwifery practice	Definition of expected professional competencies, knowledge, skills, and behaviours

**Table 5:** Reported development, validation, and measurement-property evidence across the identified assessment approaches.

Assessment Approach/Report	Development/Validation Procedures Reported	Measurement-Property Evidence Reported	Interpretation Within the Present Review
FIGO Nutrition Checklist—Killeen et al. [4]	Review and clinical discussion of the evidence-based FIGO Nutrition Checklist as a resource to support nutrition counselling during pregnancy.	The publication did not report a comprehensive psychometric evaluation of the FIGO Nutrition Checklist as a measurement instrument.	Provides evidence relevant to the clinical use of the FIGO Nutrition Checklist for nutritional-risk identification and counselling support; it was not interpreted as a patient-experience measure.
FIGO Nutrition Checklist—Jacob et al. [21]	Evaluation of the existing FIGO Nutrition Checklist among women and healthcare practitioners in preconception and early-pregnancy care.	Acceptability, feasibility, and implementation-related evidence was reported rather than conventional reliability, construct-validity, or responsiveness testing.	Provides implementation evidence concerning the FIGO Nutrition Checklist; acceptability and feasibility were distinguished from comprehensive psychometric validation.
FIGO Nutrition Checklist—Grammatikopoulou et al. [22]	Existing FIGO Nutrition Checklist applied to assess dietary quality, dietary behaviours, and nutritional risk among pregnant women.	No additional comprehensive psychometric evaluation of the checklist was reported within the included application study.	Represents a clinical application of the existing nutritional-risk screening tool rather than an instrument-development or psychometric-validation study.
Nankumbi et al. [9]	Qualitative study using in-depth interviews and structured non-participant observations of group and one-to-one nutrition education.	Conventional psychometric properties were not applicable because the approach was not a standardized measurement instrument.	Provides qualitative and direct observational evidence concerning nutrition-education and counselling delivery; it was interpreted according to this methodological purpose rather than psychometrically ranked against standardized instruments.
Kumbiley et al. [10]	Questionnaire items were adapted from previous studies and supplemented by author-developed items; content validity was assessed by nutritionists and behavioural scientists, and the questionnaire was pre-tested in five non-participant nurses/midwives.	Content-validation and pre-testing procedures were reported; no comprehensive reliability, responsiveness, or broader psychometric evaluation was reported.	Provides preliminary development and content-related evidence for a provider-focused assessment of nutrition-care practices, but insufficient evidence for comprehensive psychometric appraisal.
Masesane et al. [11]	Questionnaire adapted from previously validated questions and aligned with WHO antenatal-care recommendations; content and face validity were assessed by relevant experts, and pilot testing involving 5% of the study sample was undertaken.	Content/face-validity and pilot reliability assessment were reported; no quantitative reliability coefficient was reported.	Provides development and validation evidence for a provider-focused questionnaire combined with observational assessment, but insufficient evidence for comprehensive psychometric appraisal.
Arrish et al. [23]	Cross-sectional web-based questionnaire assessing midwives’ nutrition knowledge, attitudes, confidence, and educational practices.	No comprehensive psychometric evaluation or responsiveness evidence was reported in the included publication.	Primarily provides descriptive assessment of provider knowledge, attitudes, confidence, and practice; it was interpreted according to this intended purpose rather than as a patient-reported measurement instrument.
Olloqui-Mundet et al. [24]	Questionnaire based on previous work; expert-judgement assessment by six experts addressed sufficiency, clarity, coherence, and relevance; an 80% criterion was applied, the questionnaire was reduced from 53 to 46 items, and it was subsequently pilot-tested in 10 midwives.	Content-validation and pilot-testing evidence were reported; no reliability coefficient or responsiveness assessment was reported.	Provides structured content-validation evidence for a provider-focused nutrition-education questionnaire, but not comprehensive psychometric evaluation.
ICM Essential Competencies for Midwifery Practice [5]	International professional competency framework developed to define expected standards, knowledge, skills, and behaviours for midwifery practice, including nutrition-related competencies.	Conventional psychometric properties such as internal consistency, measurement error, and responsiveness are not directly applicable to its intended function as a professional competency framework.	Provides standards relevant to professional competence and nutrition-related midwifery practice; it was interpreted according to its framework function and was not psychometrically ranked against questionnaires or screening instruments.

**Table 6:** Provisional candidate domains for future patient-reported assessment of midwife-led perinatal nutrition counselling.

Provisional Candidate Domain	Evidence Informing Identification
1. Clarity and comprehensibility of nutrition-related communication	Qualitative evidence concerning women’s need for understandable, relevant nutrition information and broader principles of woman-centred communication [31,32].
2. Individualization and practical applicability of dietary guidance	Women valued counselling tailored to individual circumstances and everyday challenges rather than standardized dietary information [31,32].
3. Responsiveness to women’s clinical, cultural, and personal circumstances	Evidence supporting personalized nutrition counselling and responsiveness to individual needs and circumstances [31,32,33,34].
4. Involvement in nutrition-related decision-making	Qualitative evidence emphasizing women’s engagement and shared decision-making within supportive counselling relationships [31,32].
5. Continuity and consistency of counselling support	Women identified continuity and ongoing support as relevant characteristics of nutrition counselling, while provider evidence highlighted organizational constraints affecting delivery [31,32,33,34].
6. Supportive and non-judgmental counselling relationship	Qualitative evidence emphasized supportive therapeutic relationships and counselling responsive to women’s concerns and needs [31,32].
7. Women’s perceived support for understanding and acting on nutrition-related recommendations	Cross-cutting synthesis of communication, practical guidance, behavioural support, and women’s engagement with nutrition recommendations [5,9,11,24,25,31,32,33,34].

## Data Availability

The original contributions presented in this study are included in the article/Supplementary Material. Further inquiries can be directed to the corresponding author.

## Supplementary Materials

The following supporting information can be downloaded at: https://www.mdpi.com/article/10.3390/nursrep16090327/s1, Supplementary File: Full Electronic Search Strategies and Eligibility Criteria.

## Public Involvement Statement

No public involvement in any aspect of this research.

## Guidelines and Standards Statement

This manuscript was prepared in accordance with the PRISMA Extension for Scoping Reviews (PRISMA-ScR) reporting guideline and methodological guidance from the Joanna Briggs Institute (JBI) for scoping reviews.
