# Supplementary material for: Mapping Assessment Approaches to Midwife-Led Perinatal Nutrition Counselling: A Scoping Review of Existing Instruments, Competency Frameworks, and Measurement Gaps
Source: Nurs Rep. 2026 Sep 7;16(9):327. doi: 10.3390/nursrep16090327 (PMC13610695; doi:10.3390/nursrep16090327)
Supplement: Supplementary file 1 [file nursrep-16-00327-s001.zip › nursrep-4472343-supplementary.pdf]

Supplementary File . Full Electronic Search Strategies and Eligibility Criteria

Review title: Assessing the Quality of Midwife-Led Perinatal Nutrition Counseling: A Scoping Review of Existing Instruments, Competency Frameworks, and Measurement Gaps

Search coverage: Database inception to 1 July 2026.

Search approach: Two complementary searches were specified for each database. Search A targeted instruments, competency frameworks, questionnaires, checklists, screening tools, and other assessment approaches used in nutrition counselling within midwifery and perinatal care. Search B was a sensitivity-oriented patient-experience/counselling-quality search and broadened the third concept to patient-reported, patient-experience, satisfaction, person-centred and woman-centred care, shared decision-making, counselling-quality, and related terms to maximize retrieval of potentially relevant experience-based assessment approaches. Some broader outcome- or behaviour-related indexing terms were retained in the electronic search solely to improve search sensitivity; their use did not imply conceptual equivalence between patient-reported experience measures (PREMs) and patient-reported outcome measures (PROMs). Eligibility and synthesis remained focused on the predefined review question and nutrition-specific assessment within midwifery or perinatal care.

Database-specific controlled vocabulary and free-text syntax were used as applicable. No geographical restrictions were applied. Searches were limited to English-language publications.

A1. PubMed/MEDLINE

MeSH terms used: "Midwifery"[Mesh]; "Maternal Health Services"[Mesh]; "Nutritional Sciences"[Mesh]; "Nutrition Therapy"[Mesh]; "Surveys and Questionnaires"[Mesh]; "Patient Reported Outcome Measures"[Mesh]; and "Patient Satisfaction"[Mesh].

Search A – Core instrument/framework search

(("Midwifery"[Mesh] OR midwi*[tiab] OR "Maternal Health Services"[Mesh] OR "maternity care"[tiab] OR antenatal[tiab] OR prenatal[tiab] OR perinatal[tiab] OR postpartum[tiab] OR postnatal[tiab]) AND (("Nutritional Sciences"[Mesh] OR "Nutrition Therapy"[Mesh] OR nutrition*[tiab] OR diet*[tiab]) AND (counsel*[tiab] OR educat*[tiab] OR advice[tiab] OR guidance[tiab])) AND ("Surveys and Questionnaires"[Mesh] OR instrument*[tiab] OR questionnaire*[tiab] OR tool*[tiab] OR checklist*[tiab] OR framework*[tiab] OR competenc*[tiab] OR assess*[tiab] OR evaluat*[tiab] OR measur*[tiab] OR screen*[tiab]))

Search B – Patient-experience/counselling-quality sensitivity search

(("Midwifery"[Mesh] OR midwi*[tiab] OR "Maternal Health Services"[Mesh] OR "maternity care"[tiab] OR antenatal[tiab] OR prenatal[tiab] OR perinatal[tiab] OR postpartum[tiab] OR postnatal[tiab]) AND (("Nutritional Sciences"[Mesh] OR "Nutrition Therapy"[Mesh] OR nutrition*[tiab] OR diet*[tiab]) AND (counsel*[tiab] OR educat*[tiab] OR advice[tiab] OR guidance[tiab])) AND ("Patient Reported Outcome Measures"[Mesh] OR "Patient Satisfaction"[Mesh] OR "patient experience"[tiab] OR "patient-reported"[tiab] OR "woman-centred"[tiab] OR "woman-centered"[tiab] OR "person-centred"[tiab] OR "person-centered"[tiab] OR "shared decision making"[tiab] OR "shared decision-making"[tiab] OR "counselling quality"[tiab] OR "counseling quality"[tiab] OR "behaviour change"[tiab] OR "behavior change"[tiab] OR satisfaction[tiab]))

A2. Scopus

Search A – Core instrument/framework search

TITLE-ABS-KEY((midwi* OR "maternity care" OR antenatal OR prenatal OR perinatal OR postpartum OR postnatal) AND ((nutrition* OR diet*) W/3 (counsel* OR educat* OR advice OR guidance)) AND (instrument* OR questionnaire* OR tool* OR checklist* OR framework* OR competenc* OR assess* OR evaluat* OR measur* OR screen*)) AND (LIMIT-TO(LANGUAGE, "English"))

Search B – Patient-experience/counselling-quality sensitivity search

TITLE-ABS-KEY((midwi* OR "maternity care" OR antenatal OR prenatal OR perinatal OR postpartum OR postnatal) AND ((nutrition* OR diet*) W/3 (counsel* OR educat* OR advice OR guidance)) AND ("patient experience" OR "patient-reported" OR "woman-centred" OR "woman-centered" OR "person-centred" OR

"person-centered" OR "shared decision making" OR "shared decision-making" OR "counselling quality" OR "counseling quality" OR "behaviour change" OR "behavior change" OR satisfaction)) AND (LIMIT-TO(LANGUAGE, "English"))

A3. Web of Science Core Collection

Search A – Core instrument/framework search

TS=((midwi* OR "maternity care" OR antenatal OR prenatal OR perinatal OR postpartum OR postnatal) AND ((nutrition* OR diet*) NEAR/3 (counsel* OR educat* OR advice OR guidance)) AND (instrument* OR questionnaire* OR tool* OR checklist* OR framework* OR competenc* OR assess* OR evaluat* OR measur* OR screen*)) AND LA=(English)

Search B – Patient-experience/counselling-quality sensitivity search

TS=((midwi* OR "maternity care" OR antenatal OR prenatal OR perinatal OR postpartum OR postnatal) AND ((nutrition* OR diet*) NEAR/3 (counsel* OR educat* OR advice OR guidance)) AND ("patient experience" OR "patient-reported" OR "woman-centred" OR "woman-centered" OR "person-centred" OR "person-centered" OR "shared decision making" OR "shared decision-making" OR "counselling quality" OR "counseling quality" OR "behaviour change" OR "behavior change" OR satisfaction)) AND LA=(English)

A4. CINAHL (EBSCOhost)

Search A – Core instrument/framework search

((MH "Midwifery+") OR TX midwi* OR TX "maternity care" OR TX antenatal OR TX prenatal OR TX perinatal OR TX postpartum OR TX postnatal) AND (((MH "Nutrition+") OR TX nutrition* OR TX diet*) AND (TX counsel* OR TX educat* OR TX advice OR TX guidance)) AND ((MH "Questionnaires+") OR TX instrument* OR TX questionnaire* OR TX tool* OR TX checklist* OR TX framework* OR TX competenc* OR TX assess* OR TX evaluat* OR TX measur* OR TX screen*)

Search B – Patient-experience/counselling-quality sensitivity search

((MH "Midwifery+") OR TX midwi* OR TX "maternity care" OR TX antenatal OR TX prenatal OR TX perinatal OR TX postpartum OR TX postnatal) AND (((MH "Nutrition+") OR TX nutrition* OR TX diet*) AND (TX counsel* OR TX educat* OR TX advice OR TX guidance)) AND ((MH "Patient Satisfaction+") OR TX "patient experience" OR TX "patient-reported" OR TX "woman-centred" OR TX "woman-centered" OR TX "person-centred" OR TX "person-centered" OR TX "shared decision making" OR TX "shared decision-making" OR TX "counselling quality" OR TX "counseling quality" OR TX "behaviour change" OR TX "behavior change" OR TX satisfaction)

A5. Embase

Search A – Core instrument/framework search

('midwifery'/exp OR midwi*:ti,ab,kw OR 'maternity care':ti,ab,kw OR antenatal:ti,ab,kw OR prenatal:ti,ab,kw OR perinatal:ti,ab,kw OR postpartum:ti,ab,kw OR postnatal:ti,ab,kw) AND (('nutrition'/exp OR nutrition*:ti,ab,kw OR diet*:ti,ab,kw) AND (counsel*:ti,ab,kw OR educat*:ti,ab,kw OR advice:ti,ab,kw OR guidance:ti,ab,kw)) AND ('questionnaire'/exp OR instrument*:ti,ab,kw OR questionnaire*:ti,ab,kw OR tool*:ti,ab,kw OR checklist*:ti,ab,kw OR framework*:ti,ab,kw OR competenc*:ti,ab,kw OR assess*:ti,ab,kw OR evaluat*:ti,ab,kw OR measur*:ti,ab,kw OR screen*:ti,ab,kw) AND [english]/lim

Search B – Patient-experience/counselling-quality sensitivity search

('midwifery'/exp OR midwi*:ti,ab,kw OR 'maternity care':ti,ab,kw OR antenatal:ti,ab,kw OR prenatal:ti,ab,kw OR perinatal:ti,ab,kw OR postpartum:ti,ab,kw OR postnatal:ti,ab,kw) AND (('nutrition'/exp OR nutrition*:ti,ab,kw OR diet*:ti,ab,kw) AND (counsel*:ti,ab,kw OR educat*:ti,ab,kw OR advice:ti,ab,kw OR guidance:ti,ab,kw)) AND ('patient reported outcome'/exp OR 'patient satisfaction'/exp OR 'patient experience':ti,ab,kw OR 'patient-reported':ti,ab,kw OR 'woman-centred':ti,ab,kw OR 'woman-centered':ti,ab,kw OR 'person-centred':ti,ab,kw OR 'person-centered':ti,ab,kw OR 'shared decision making':ti,ab,kw OR 'shared decision-making':ti,ab,kw OR

'counselling quality':ti,ab,kw OR 'counseling quality':ti,ab,kw OR 'behaviour change':ti,ab,kw OR 'behavior change':ti,ab,kw OR satisfaction:ti,ab,kw) AND [english]/lim

A6. Additional searching

Reference lists of included studies and relevant review articles were screened manually for additional eligible publications. Grey-literature sources were also considered through the websites of major international organisations and professional bodies relevant to maternal health, nutrition, and midwifery, including the World Health Organization (WHO), International Confederation of Midwives (ICM), International Federation of Gynecology and Obstetrics (FIGO), and Nursing and Midwifery Board of Ireland (NMBI).

A7. Eligibility criteria applied to the retrieved evidence

Eligibility criteria were established a priori according to the Population–Concept–Context (PCC) framework and the objectives of the review.

Inclusion criteria

- Studies or documents describing, developing, validating, applying, or evaluating instruments, competency frameworks, questionnaires, screening tools, checklists, or other assessment approaches related to nutrition counselling, nutrition education, nutrition-related competencies, counselling quality, or women's experiences of nutrition counselling within midwifery or perinatal care.
- Original quantitative, qualitative, and mixed-methods studies; methodological studies describing instrument development or validation; observational studies; consensus statements; clinical practice guidelines; competency frameworks; and reports from professional organizations.
- Publications involving midwives, pregnant women, postpartum women, or other healthcare professionals providing nutrition counselling during the perinatal period.
- For broad professional competency frameworks, explicitly identifiable nutrition- or nutrition-counselling-related competencies that could be extracted and mapped against the review objectives.
- No geographical restrictions were applied. Publications were required to be available in English.

Exclusion criteria

- Studies focused exclusively on clinical nutritional interventions, dietary supplementation, or nutritional outcomes without evaluating any aspect of nutrition counselling or its assessment.
- Studies addressing general health education without a nutrition component.
- Editorials, conference abstracts, letters, commentaries, protocols, and publications without sufficient methodological information.
- General midwifery competency or professional assessment frameworks without sufficiently specific nutrition-related content.
- Generic patient-experience, communication, shared decision-making, or person-centred care instruments without a specific nutrition-counselling component in midwifery or perinatal care. Such broader instruments were eligible only when applied to, or explicitly developed for, nutrition counselling within the predefined Concept and Context of the review.

A8. Reporting note

The database-specific syntax above reproduces the electronic search concepts used in the review and is provided to improve transparency and reproducibility. The PubMed/MEDLINE MeSH terms are listed explicitly above; equivalent controlled vocabulary was used in CINAHL and Embase where applicable, together with database-specific free-text syntax. The review identified 186 records across the electronic database searches before duplicate removal. Database-specific retrieval counts were not retained and therefore are not reported separately.
